# Bacterial communities associated with *Acrobeles complexus* nematodes recovered from tomato crops in South Africa

**DOI:** 10.1371/journal.pone.0304663

**Published:** 2024-06-06

**Authors:** Ebrahim Shokoohi, Ricardo A. R. Machado, Peter Masoko

**Affiliations:** 1 Department of Biochemistry, Microbiology, and Biotechnology, University of Limpopo, Sovenga, South Africa; 2 Experimental Biology, Institute of Biology, University of Neuchatel, Neuchatel, Switzerland; Bayero University Kano, NIGERIA

## Abstract

The productivity of agricultural ecosystems is heavily influenced by soil-dwelling organisms. To optimize agricultural practices and management, it is critical to know the composition, abundance, and interactions of soil microorganisms. Our study focused on *Acrobeles complexus* nematodes collected from tomato fields in South Africa and analyzed their associated bacterial communities utilizing metabarcoding analysis. Our findings revealed that *A*. *complexus* forms associations with a wide range of bacterial species. Among the most abundant species identified, we found *Dechloromonas* sp., a bacterial species commonly found in aquatic sediments, *Acidovorax temperans*, a bacterial species commonly found in activated sludge, and *Lactobacillus ruminis*, a commensal motile lactic acid bacterium that inhabits the intestinal tracts of humans and animals. Through principal component analysis (PCA), we found that the abundance of *A*. *complexus* in the soil is negatively correlated with clay content (*r* = -0.990) and soil phosphate levels (*r* = -0.969) and positively correlated with soil sand content (*r* = 0.763). This study sheds light on the bacterial species associated to free-living nematodes in tomato crops in South Africa and highlights the occurrence of various potentially damaging and beneficial nematode-associated bacteria, which can in turn, impact soil health and tomato production.

## Introduction

Free-living nematodes and their associated bacterial communities can positively impact crop health and productivity [1]. In addition, Free-living nematodes can improve soil health, nutrient cycling and even control pests [1]. Thus, understanding and characterizing the biodiversity of free-living nematodes and their associated bacterial communities in crop ecosystems is crucial for developing sustainable agriculture practices [2–5].

Various feeding groups of nematodes have been reported in association with tomato fields in Limpopo Province, South Africa [4, 6]. *Acrobeles complexus* is a free-living bacterivore nematode that has been found in tomato fields in Dalmada, Limpopo Province. Microbiomes, including bacteria, fungi, viruses, and archaea, have been studied in a various range of animals, which play an important role in their development [7]. McQueen et al. [8] studied internal and external microbiomes associated with *Plectus murrayi*, showing that Comamonadaceae, followed by Pseudomonaceae families, are the most dominant internal and external bacteria. The phylum Proteobacteria (46.45% of the bacterial taxa) was the most abundant bacterial phylum in Monhysteridae, Rhabditidae and Panagrolaimidae families of free-living bacterivores nematodes, followed by Bacteroidetes (20.47%), Actinobacteria (5.97%), Firmicutes (5.67%), and Cyanobacteria (2.47%) [9].

The intricate and complex network of soil microbial communities plays a vital role in maintaining a healthy ecosystem. These communities are responsible for various ecosystem services such as recycling nutrients, sequestering carbon, retaining water, and promoting plant growth and defense [5]. By delving deeper into the interactions between plants, nematodes, and microbes in both natural and agricultural ecosystems, we can develop innovative biotechnological tools to manage soil fertility and achieve a high-yield food production system [10, 11]. Therefore, microbiome study is crucial for agricultural and environmental ecosystems. So far, none of the Rhabditida free-living bacterivores nematodes microbiomes has been studied in South Africa, except for *Zeldia punctata*. Microbiome associated with *Z*. *punctata* from maize fields in Limpopo Province, South Africa, was studied, indicating the dominance of *Pseudomonas* and *Lactobacillus* [12]. Yet, there is no information on the microbiome associated with *A*. *complexus* in South Africa, and therefore, the aims of the study were 1) to study the microbiome of *A*. *complexus* using 16S rDNA metagenomics by Illumina Miseq technology and 2) to study the relationship of *A*. *complexus* with selected soil parameters using multivariate analysis.

## Materials and methods

### Nematode sampling and processing

**Fig 1:**
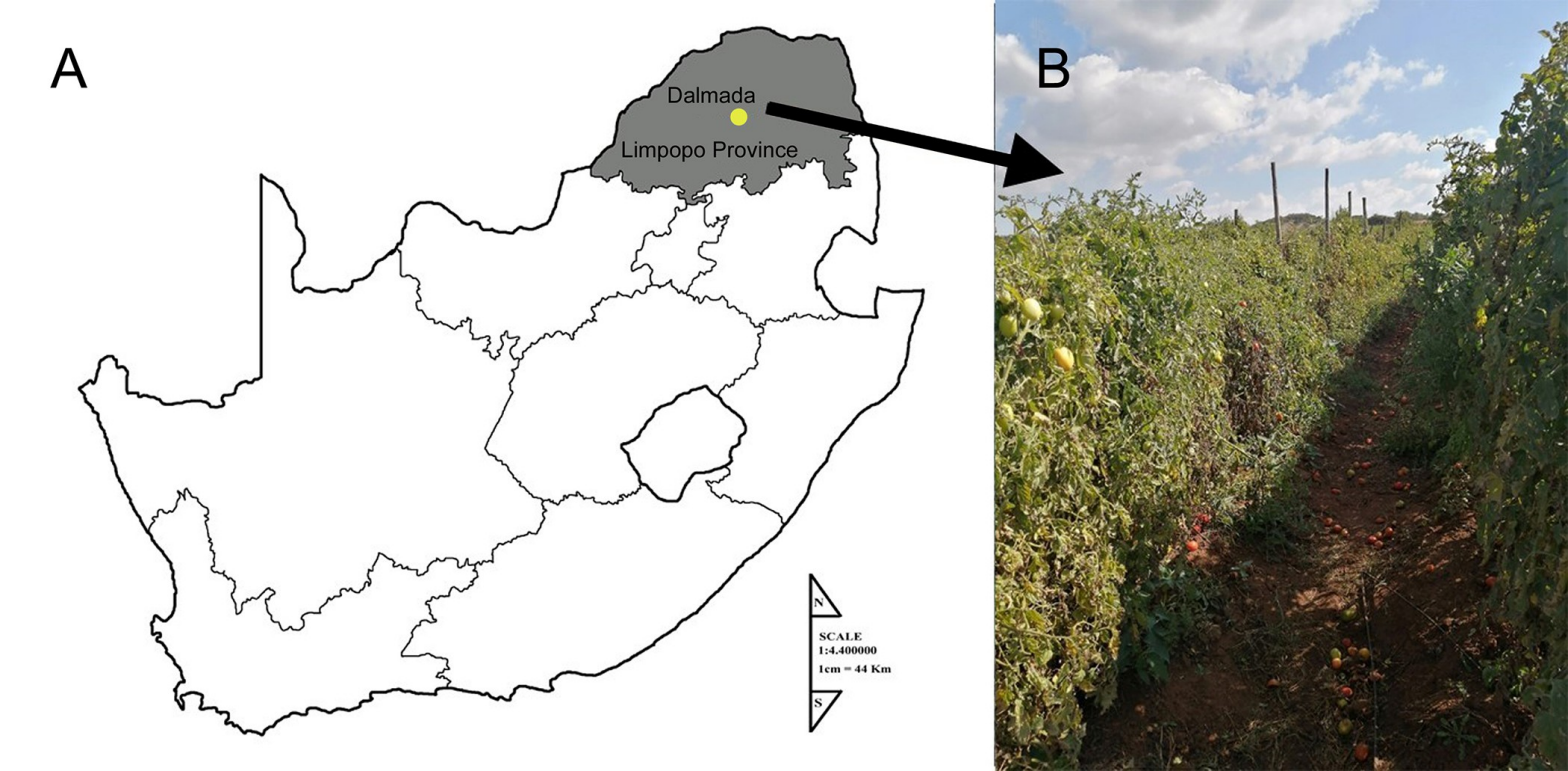
Sampling of *A*. *complexus* from Dalmada, Limpopo Province, South Africa. A, B: Map of South Africa indicating the approximate location of the tomato field where soil samples to isolate *A*. *complexus* nematodes were collected.

The specimens of *A*. *complexus* were obtained from soil samples that were collected randomly from a tomato field situated in Dalmada, Limpopo Province, South Africa (GPS coordinates of the field: S 23° 53.798’, E 029° 32.773’) (Fig 1). The map was made using paint.net version 5.0.13, which is freely available. The base map was built using the freely available 4.01 Biomes in Africa Map. Three soil cores, weighing approximately 500 g each, were collected at intervals of about 10 m from each other, at depth of 30 cm. Nematodes were extracted from the soil samples through the modified tray technique [13]. For this, 500 g of soil was separately transferred into a plastic baskets. Nematodes were then recovered after 72 h of incubation. The samples were subsequently studied using a Zeiss light microscope at the Aquaculture Research Unit. The *A*. *complexus* specimens were selectively isolated based on their morphological characteristics. The following morphological traits were taken into consideration: specimens with long labial probolae, terminal bulb with valve, and conical tail. Five specimens per sample were subjected to molecular and microbiome analysis, while the rest of specimens were used for detailed morphological characterization and microscopic observations. To preserve *A*. *complexus* specimens, they were first immersed in hot 4% formaldehyde and then transferred to glycerin [14]. Permanent glass slides were utilized to mount them for observation through an Olympus light microscope (Japan) with differential interference contrast optics (DIC). LM photos of the slides were captured and refined using Adobe® Photoshop® CS.

### *A*. *complexus* molecular identification

#### DNA extraction

The present investigation employed the Chelex method, as described by Straube and Juen [15], for the extraction of DNA. Three specimens of *A*. *complexus* were manually selected and transferred to a 1.5-ml Eppendorf tube preloaded with 5 μl of water, with a total of three independent samples being processed. The nematodes were mechanically crushed and mixed using a fine-tipped needle and vortexing, respectively. Subsequently, 60 microliters of 10% Chelex® 50 and 5 μl of proteinase K were added to each microcentrifuge tube containing the crushed nematodes and mixed. Afterward, the nematode lysates were subjected to an incubation period of 1 hour at 65°C, followed by incubation at 95°C for 10 minutes to deactivate the proteinase K. Finally, the lysates were centrifuged at 16,000 rpm for 2 minutes, following the protocols described by Shokoohi and Eisenback [16]. The resulting supernatants were collected and stored at -20°C for further analyses.

#### Ribosomal RNA gene amplification and sequencing

For molecular identification, the 28S ribosomal RNA gene was amplified by PCR, and the obtained PCR products were sequenced by Sanger sequencing. PCR was carried out using D2A (5′-ACAAGTACCGTGAGGGAAAGTTG–3′) and D3B (5′-TCGGAAGGAACCAGCTACTA–3′) primers [17]. PCR reactions were conducted using 5 μl of DNA extracts, 12.5 μl of 2X PCR Master Mix Red (NEB, USA), 1 μl of each primer (10 pmol μl−1), and 5.5 μl of ddH2O to make a final volume of 25 μl. The following cycling program was used: initial denaturation for 3 minutes at 94°C, 37 cycles of denaturation for 45 seconds at 94°C, annealing temperatures at 56°C, extension for 45 seconds at 72°C, and a final extension step of 6 minutes at 72°C, followed by a hold at 4°C. Once amplification was completed, 4 μl of PCR products from each tube were loaded onto a 1.5% agarose gel in TBE buffer (40 mM Tris, 40 mM boric acid, and 1 mM EDTA) to visualize PCR products. The bands were stained with SafeView (abm, Canada) and then viewed and captured on a UV transilluminator. Finally, the PCR products were purified for Sanger sequencing at Inqaba Biotec (Pretoria, South Africa). The obtained sequences were manually curated, and all sequences were deposited in the National Center for Biotechnology Information (NCBI) database under the accession numbers: OR889721, OR889722, and OR889724.

#### Phylogenetic analysis

The phylogenetic analysis was carried out using the obtained 28S rRNA gene sequences using the maximum likelihood method. Prior to this analysis, best-fit substitution model analyses were conducted to ensure accuracy. Trees were recustructed using the Neighbour-joining (NJ) method with 1000 bootstrap values replicates. A discrete Gamma distribution model with 5 categories was used for the tree analysis. The phylogenetic tree was recustructed using MEGA 11 [18], and the phylogenetic trees were edited using the Interactive Tree of Life (version 3.5.1) developed by Chevenet et al. [19] and Letunic and Bork [20].

#### *A*. *complexus* microbiome analyses

To characterize the microbiome associated with *A*. *complexus* nematodes, the following procedure was carried out. First, the nematodes were disinfected with a 1% sodium hypochlorite solution for three minutes, followed by a thorough washing with distilled water, as per the methodology recommended by Esser [21]. Subsequently, the ZymoBIOMICS™ DNA Miniprep Kit was used to extract total DNA, following the manufacturer’s instructions. The quality and quantity of the extracted DNA were determined through agarose gel electrophoresis and a NanoDrop 2000C spectrophotometer. To amplify the V3-V4 regions of the bacterial 16S gene sequences, universal bacteria primers 341f (5′-CCTACGGGNGGCWGCAG-3′) and 785r (5′-GACTACHVGGGTATCTAATCC-3′) were used, as recommended by Herlemann et al. [22]. The PCR reaction mixture included 10–15 ng of the DNA template, 12.5 μl of 2X PCR Master Mix Red, 1 μl of each primer (10 pmol μl′1), and ddH_2_O to make up a final volume of 25 μl. The DNA amplification was carried out using an Eppendorf Mastercycler Gradient, with a temperature program consisting of initial denaturation at 94°C for three minutes, followed by 37 cycles of denaturation for 45 seconds at 94°C, annealing at 50°C, extension at 72°C for 45 seconds, and a final extension step at 72°C for six minutes, followed by a temperature hold at 4°C.

The DNA bands were evaluated by staining a 1.5% agarose gel with SafeView and visualizing them on a UV transilluminator. The resulting amplicons were purified from the gel and analyzed using 2% agarose gel analysis with SafeView Classic (abm, Canada). The purified DNA was then sequenced on an Illumina MiSeq platform using a MiSeq v3 (600 cycles) kit. Each sample generated 20 megabytes of data in the form of paired-end reads that were 300 base pairs long. The data were deposited in the NCBI database under the following biosample accession numbers: SAMN20694755, SAMN20694756, and SAMN20694757.

#### Bioinformatics of *A*. *complexus* microbiome

The Quantitative Insights Into Microbial Ecology 2 (QIIME 2 2017.4) software was used for microbiome bioinformatics [23–25]. The paired-end reads, and manifest file were imported into QIIME2, creating a compressed paired-end-demux.qza file. The reads were pre-processed and filtered using the Divisive Amplicon Denoising Algorithm 2 (DADA2) plugin. Raw sequence data went through quality filtering with DADA2 [26] via q2‐dada2. The input read pairs for *A*. *complexus* isolate 1, isolate 2, and isolate 3 were 148652, 535252, and 211988, respectively. The sequences were then filtered to 23378, 91614, 55948 read pairs for *A*. *complexus* isolate 1, isolate 2, and isolate 3, respectively. All the variants of amplicon sequences (ASVs) were aligned using MAFFT [27] through q2-alignment. The QIIME2 feature-classifier plugin was used to assign taxonomies to the Denoised Amplicon Sequence Variants (ASVs). The phylogeny was constructed using fasttree2 [28] via q2-phylogeny. The samples were rarefied (subsampled without replacement) to 5698 sequences per sample [29], and then alpha-diversity metrics (observed features) and Faith’s phylogenetic diversity [30] were calculated using q2-diversity. The assignment of taxonomy to ASVs was done using q2-feature-classifier and Greengenes 13_8 99% OTUs reference sequence database [23, 24, 31].

#### Soil physicochemical analysis

Various soil chemical properties, such as ammonia, nitrate, and phosphate levels, were analyzed at the Aquaculture Research Unit laboratory. The assessment was done using a Hach spectrophotometric device (USA) following the company’s prescribed protocol. In addition, the pH was measured with the Thermo Scientific Orion 3 Star pH Benchtop (USA). Standard APHA [32] method and test number EPA 200.7 were used to analyze the samples for potassium (K), using Method 3120 B and EPA 200.7. The company protocol [33] was followed to analyze ammonia, total phosphate (P), and nitrate using the Cadmium reduction method. Total nitrogen (N) using test number 1.14537.0001; ammonia and ammonium using test number 1.14752.0001/1.14752.0002/1.00683.0001; and total phosphate (P) using test number 1.14848.0001 were measured. Nitrate was analyzed using the Cadmium reduction method no. 8171 of DOC316.53.01069 [33]. The results for phosphate, ammonia, and copper were obtained according to USEPA PhosVer 3 [33]. Spectrophotometry was used to analyze all soil properties. Soil texture was determined according to the method provided by van Capelle et al. [34].

#### Relationship of *A*. *complexus* and soil physicochemical variables

The correlation between the abundance of *A*. *complexus* and soil physicochemical properties was studied using Pearson correlation implemented in XLSTAT [35]. In order to examine the connection between several soil variables (e,g., pH, copper, ammonia, nitrate, phosphate, soil texture, and nematode), a principal component analysis (PCA) was performed, following the methodology of Renčo et al. [36]. The abundance of nematode genera was utilized to arrange the sites through a PCA conducted with XLSTAT [35]. Supplementary variables, representing soil properties, were used to identify the correlation between the abundances of *A*. *complexus*. Each variable was assigned a score based on the principal components, and the first two components (PC1 and PC2) were utilized to create a two-dimensional plot based on the eigenvalues given by the XLSTAT software.

## Result

### *A*. *complexus* diagnosis

**Fig 2:**
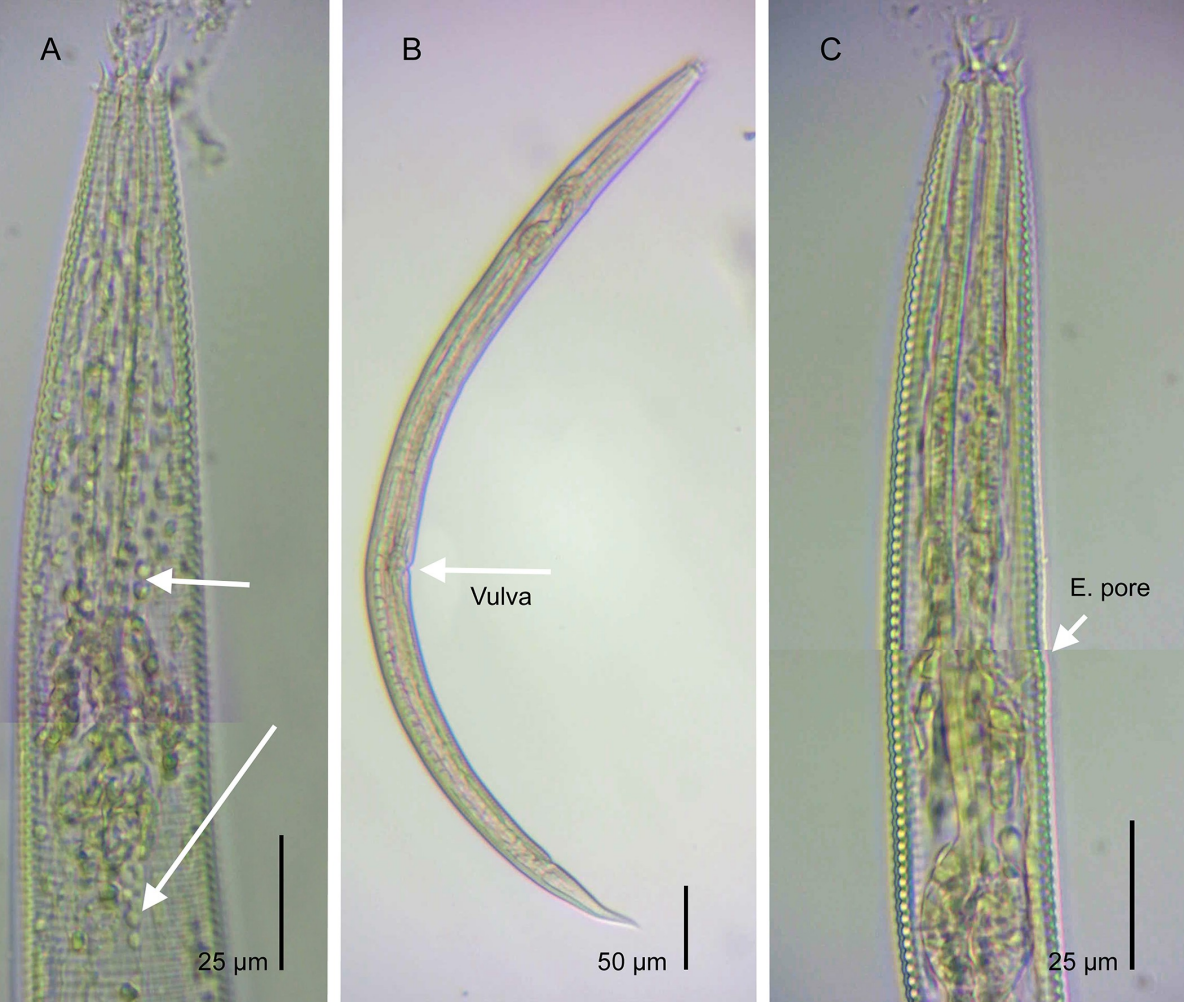
Light micrographs of *A*. *complexus* (Thorne, 1925). A: Anterior end (arrow pointing to the bacterial isolation inside body), B: Entire female (arrow pointing to vulva), C: Anterior end (arrow pointing to excretory pore).

**Fig 3:**
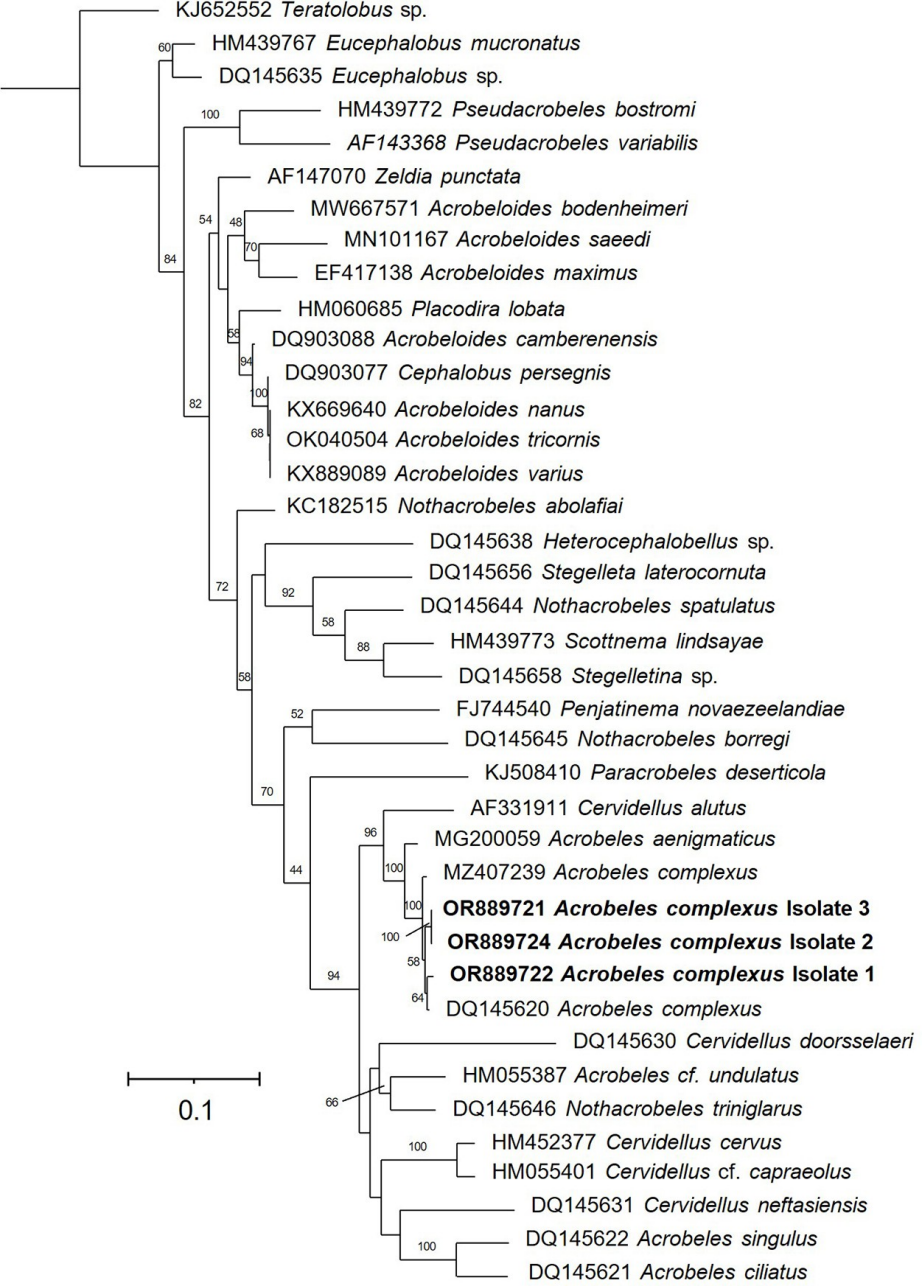
Phylogenetic tree based on ribosomal RNA gene sequences of the nematodes isolated in this study and some related species.

The *Acrobeles complexus* specimens analyzed in this study species have the following characteristics: female body length (L) between 620–663 μm; a = 17.4–23.8, b = 3.9–4.3, c = 9.3–9.5, V = 53–55), its labial probolae (12–14 μm in size), which have bifurcated and rounded tines at prongs, double cuticle in both sexes, a very long post-vulval uterine sac (69–78 μm), female tail (67–71 μm, c’ = 3.7–3.9), and curved spicules (43 μm) with rounded manubrium (Fig 2 and S1 Table). The South African specimens of *A*. *complexus* examined in this study are consistent with those studied previously (Shokoohi et al. 2007). Also, there are no noticeable differences between the specimens of *A*. *complexus* collected from various habitats in South Africa. Furthermore, the isolated species from South Africa was confirmed as *A*. *complexus* through molecular analysis based on sequences of 28S rDNA (Fig 3). The phylogenetic analysis placed all *Acrobeles* in one group with 100 bootstrap value support. Additionally, *A*. *complexus* formed a group with 100 bootstrap value support. Besides, South African *A*. *complexus* showed 98% similarity with Spanish (acc. nr: MZ407239) and American (acc. nr: DQ145620) populations of *A*. *complexus*.

### Bacterial diversity analysis

**Fig 4:**
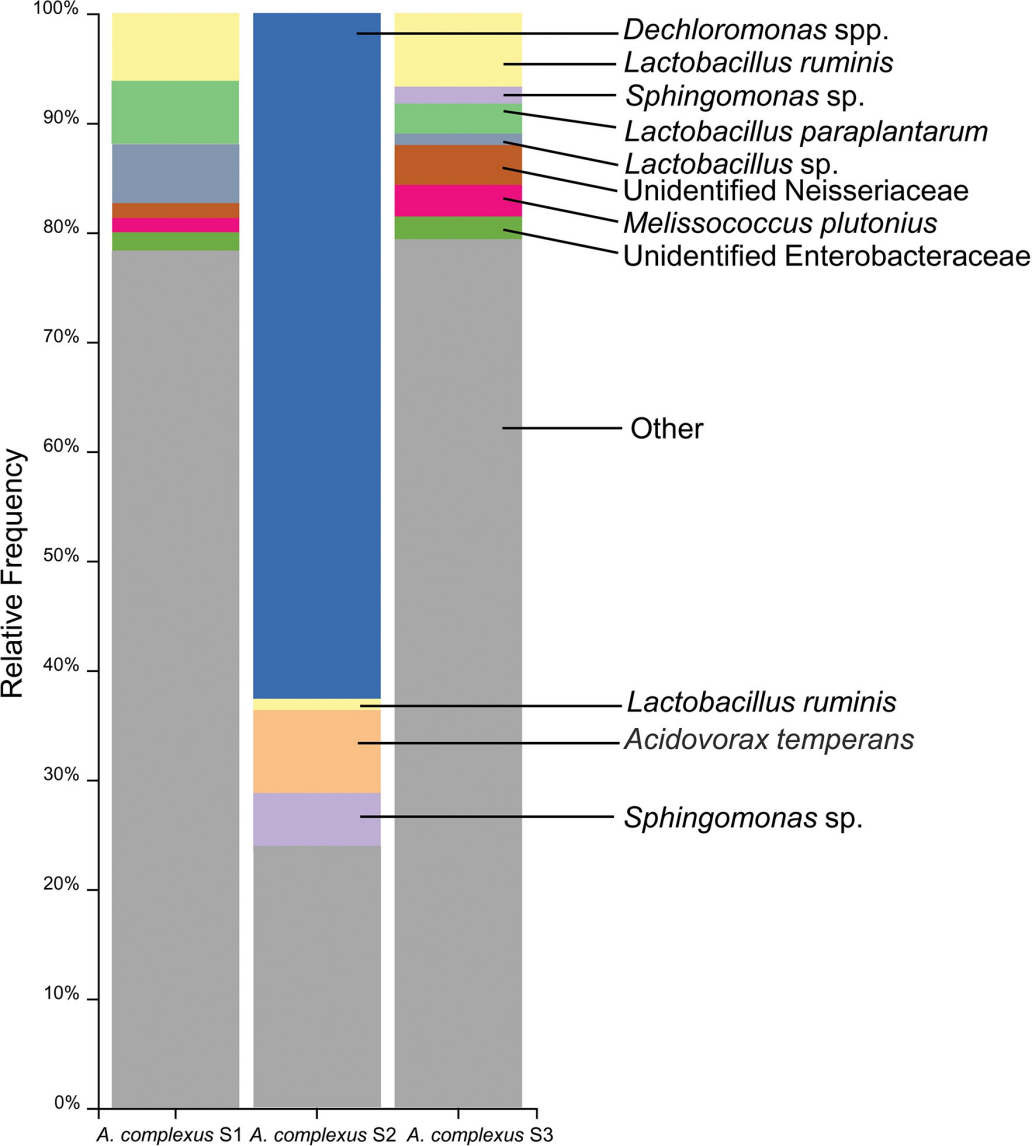
Relative frequency of the most abundant bacterial species associated with *A*. *complexus* nematodes collected from tomato fields.

After denoising and alignment of the forward and reverse reads, 359 sequences, representing 168 Amplicon Sequence Variants (ASVs), were assembled across the three biologically replicated nematode samples. Among these ASVs, we found 102, 103, and 168 ASVs in samples *A*. *complexus* S1, *A*. *complexus* S2, and *A*. *complexus* S3, respectively. According to the rarefaction plot of alpha diversity, it can be inferred that the number of counts taken during the sampling process, which amounted to 5698, is enough to encompass the diversity of all the samples studied. This conclusion can be corroborated by referring to S1 Fig, which visually represents the data. The bacterial communities’ diversity defined by Shannon entropy was highest in *A*. *complexus* S3 (7.04), intermedium in *A*. *complexus* S1 (6.21), and lowest in *A*. *complexus* S2 (4.88), as illustrated in S2 Fig. After conducting a thorough examination of the taxonomic affiliation of the representative sequences, we found that certain types of bacteria are more commonly found in association with *A*. *complexus* nematodes. Specifically, the most abundant bacteria belong to the phyla Proteobacteria, Firmicutes, Actinobacteria, and Bacteroidetes, as documented in S3 Fig. These phyla were represented by the bacterial classes Betaproteobacteria, Bacilli, Alphaproteobacteria, Actinobacteria, Gammaproteobacteria, and Clostridia (S4 Fig). Subsequently, orders Rhodocyclales, Lactobacillales, Burkholderiales, Rhizobiales, Actinomycetales, Clostridiales, Sphingomonadales, Enterobacteriales, Bacillales, and Neisseriales (S5 Fig), which in turn were represented by the families Rhodocyclaceae, Lactobacillaceae, Comamonadaceae, Sphingomonadaceae, Enterobacteriaceae, Oxalobacteraceae and Ruminococcaceae (S6 Fig). The most dominant genera were *Dechloromonas*, *Lactobacillus*, *Sphingomonas*, and *Melissococcus* (S7 Fig), and the most dominant species detected across all the *A*. *complexus* were *Dechloromonas* spp., *Acidovorax temperans*, *Lactobacillus ruminis*, *Sphingomonas* sp., *Lactobacillus paraplantarum*, and *Melissococcus plutonius* (Fig 4). Besides, several species were found in association with all three isolates (Fig 4). Among the species, *Clavibacter* (= *Corynebacterium* sp.), and *Streptomyces* were found in all three isolates of *A*. *complexus*, which are considered as plant pathogen and plant growth promoters, respectively.

### Correlation between *A*. *complexus* and soil parameter

**Fig 5:**
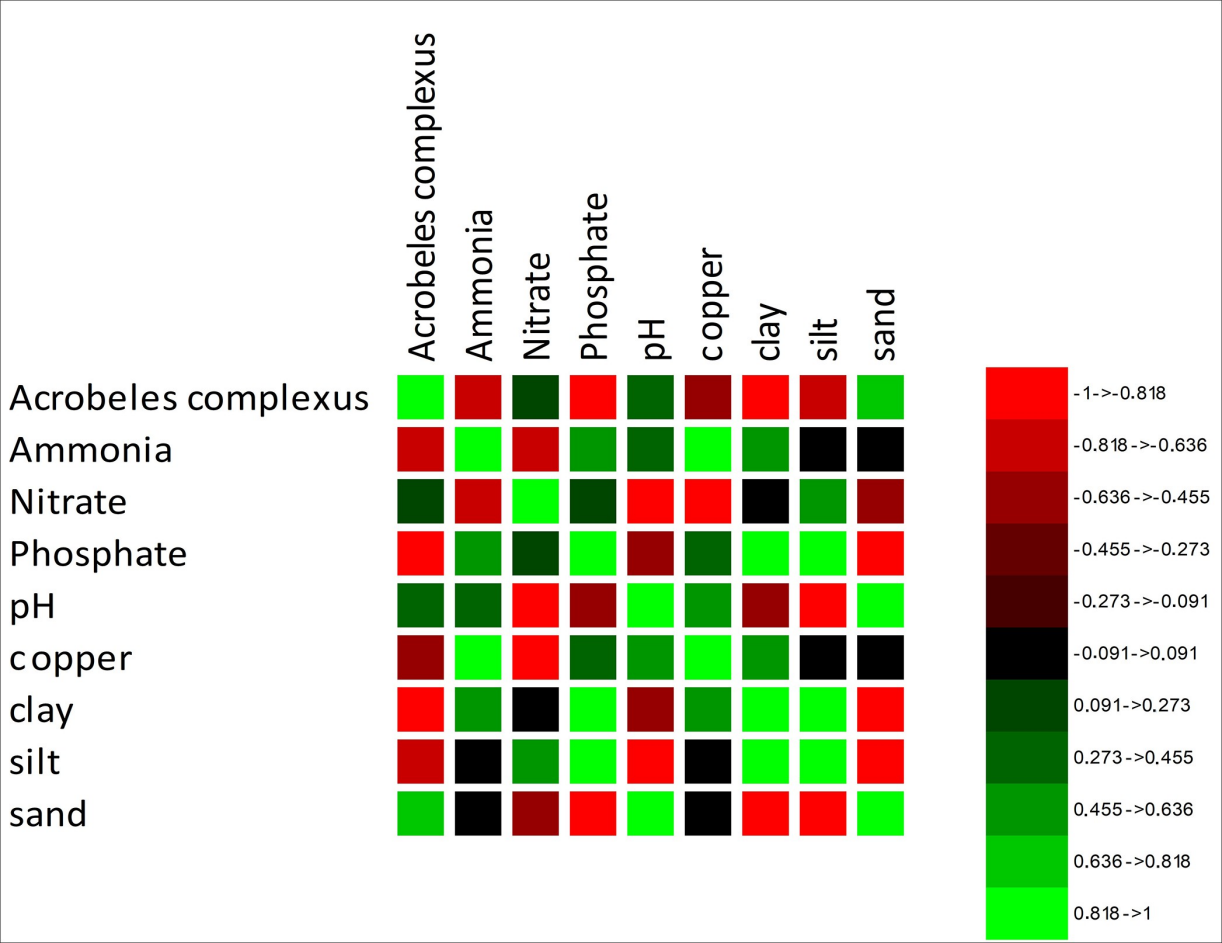
Correlation between *A*. *complexus* nematodes collected from tomato fields and selected soil parameters.

**Fig 6:**
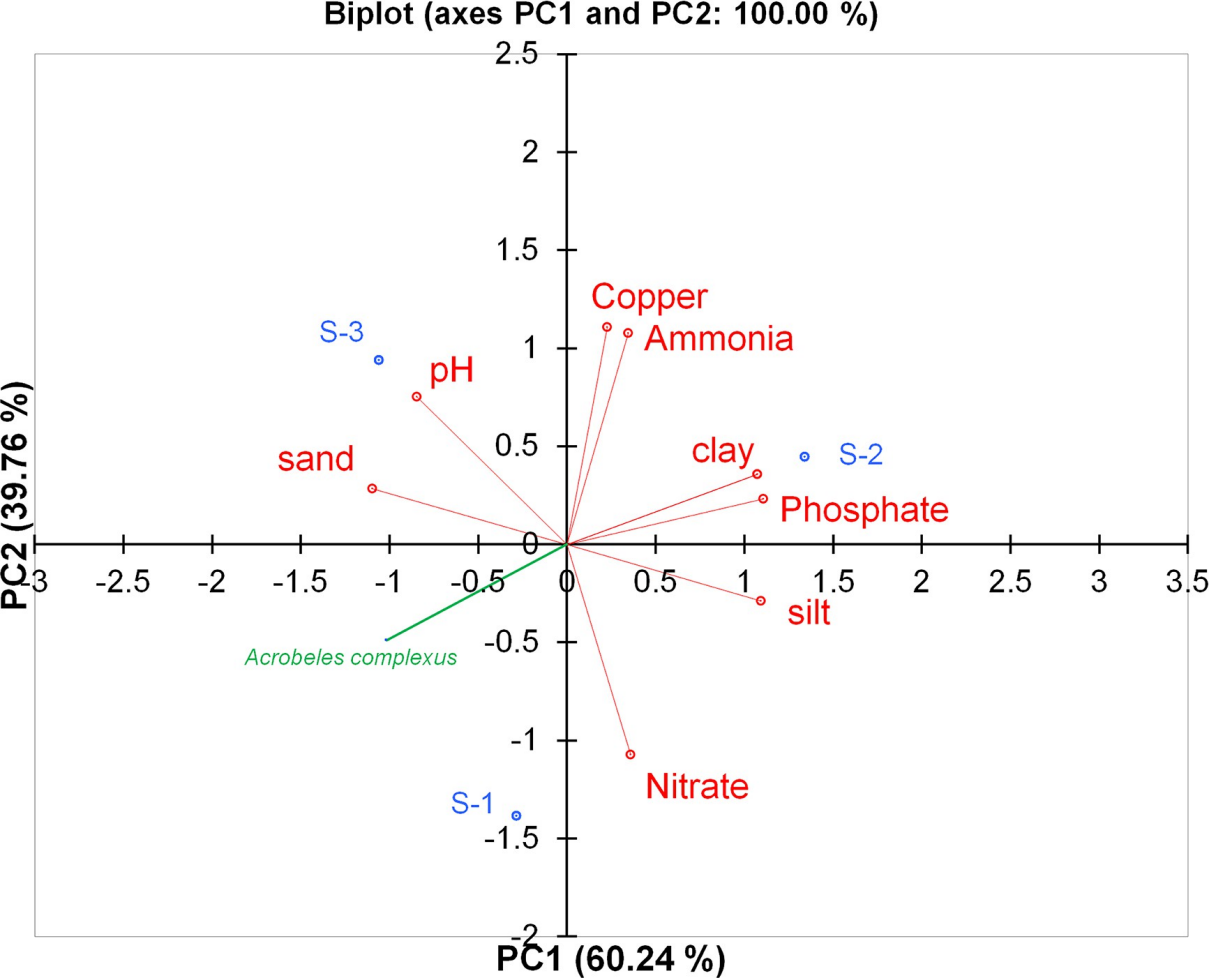
Principal component analysis between *A*. *complexus* nematodes collected from tomato fields and selected soil parameters.

The result specified a significant negative correlation (*p* < 0.05) between the abundance of *A*. *complexus* with soil clay content (*r* = -0.990) and soil phosphate levels (*r* = -0.969). On the other hand, the abundance of *A*. *complexus* was positively correlated with soil sand content (*r* = 0.763). In contrast, there were no significant correlations observed between the abundance of *A*. *complexus* and soil nitrate content (*r* = 0.126). According to the Principal Component Analysis (PCA), the variation in the distribution of *A*. *complexus* in the soil is entirely explained (100.0%) by two factors, PC1 (60.24%) and PC2 (39.76%). The analysis revealed that soil physicochemical properties significantly influenced the distribution of *A*. *complexus* in the soil (Fig 5). The study also found that *A*. *complexus* was more dominant in soils with higher sand content, pointing that in soil with more sand, more number of *A*. *complexus* exist. On the other hand, a high percentage of clay in the soil, copper, and phosphate was associated with a lower abundance of *A*. *complexus*. In addition, soil pH (*r* = 0.389) has no significant correlation with the number of *A*. *complexus* in the tomato soil of Dalmada, South Africa (Fig 6).

## Discussion

Free-living nematodes are known to feed on various sources, such as bacteria, algae, fungi, dead organisms, and living tissues. They play a crucial role in releasing nutrients that are beneficial for plant growth and also help in improving soil structure and water holding capacity. These nematodes are typically found in large numbers in soil [37]. However, free-living members of the order Rhabditida mainly feed on bacteria [38].

Regarding the *A*. *complexus*, this species is primarily found in light soils of South Africa. The same species was found in south Wales in sandy soils [39]. This species, among the bacterivores nematodes of Cephalobina, is particularly interesting due to its worldwide distribution and the complexity of its lip region and probolae [40].

The same characters were found in the present specimens of *A*. *complexus* from South Africa. Labial probolae is the main feature of this species dealing with bacterial grabbing from the environment where this species live. In fact, labial probolae act as a filter to assist the nematode with the specific size of bacteria fed by *A*. *complexus*. The morphology of this species was in agreement with what was previously found in various localities [38, 41]. The phylogenetic position of *A*. *complexus* is in agreement with the previous studies [42], in which *Acrobeles* form a group based on the 28S rDNA sequences.

Regarding the microbiome study, we comprehensively analyzed the bacterial species associated with *A*. *complexus* in this research. Our findings showed that the microbiome of *A*. *complexus* consists of a diverse range of 168 Amplicon Sequence Variants (ASVs). The microbiome is mainly composed of two dominant phyla: Proteobacteria and Firmicutes. Overall, our study provides important insights into the microbial community that exists within *A*. *complexus* and may have significant implications for future research on this organism. Among the most abundant species identified were *Dechloromonas* sp., *Acidovorax temperans*, *Lactobacillus ruminis*, *Sphingomonas* sp., and *Melissococcus plutonius*.

Soil nematodes, which are microscopic roundworms, are known to form a wide range of associations with different types of soil bacteria. These associations can vary significantly depending on the species of nematode, as well as the taxonomic identity of the bacteria involved.

According to Ogier et al. [43], *Steinernema carpocapsae* nematodes have been found to form associations with various bacterial genera, including *Pseudomonas*, *Stenotrophomonas*, *Alcaligenes*, *Achromobacter*, *Pseudochrobactrum*, *Ochrobactrum*, *Brevundimonas*, and *Delftia*. This information could be useful in further research on the interactions between nematodes and bacteria, and could lead to the enhancement of new approaches for managing nematode populations. *Acrobeloides maximus* [44] and *Zeldia punctata* [12] were two species of the family Cephalobidae, which their microbiome studied. Three bacteria, including *Ochrobactrum*, *Pedobacter*, and *Chitinophaga* were found in association with *A*. *maximus* with a high frequency of occurrence. The mentioned above bacteria were not found in *A*. *complexus* due to the different environments and locality where the species recovered. In contrast, three bacteria species, including *Pseudomonas syringae*, *Lactobacillus paraplantarum*, and *Melissococcus plutonius* were found in association with *Z*. *punctata* [12]. The same result was obtained in the present study because of the same localities where both nematode species recovered from Limpopo Province, South Africa.

*Acrobeles* nematodes are known for their non-selective feeding behavior, as they do not show any preference for specific types of bacteria. However, a comprehensive study conducted in the Netherlands revealed that *Acrobeles* species that inhabit grasslands have the most diverse bacterial communities compared to other nematode-feeding groups [45]. This finding suggests that the grassland environment provides a rich source of bacterial diversity that supports the growth and survival of *Acrobeles* nematodes. *Acrobeles* possess unique structures called labial probolae [41], which are specialized appendages that aid in the removal of bacteria from their surrounding environment. This feature assists *Acrobeles* in grabbing the bacteria from their habitat, resulting in a greater diversity of soil-dealing bacteria inside the nematode’s body. The *A*. *complexus* nematodes analyzed in this survey were linked chiefly with four bacteria, including *Dechloromonas* sp., *Acidovorax temperans*, *L*. *ruminis*, *Sphingomonas* sp., and *M*. *plutonius*.

*Dechloromonas* is a fascinating genus of bacteria that belongs to the Betaproteobacteria class. These gram-negative bacteria are generally found in soil and are recognized for their ability to degrade and detoxify a wide range of pollutants, including chlorinated compounds. *Dechloromonas* is an important player in the nitrogen cycle, as it is capable of converting nitrate to nitrogen gas. Despite being ubiquitous, these bacteria are still not fully understood, and ongoing research is shedding new light on their unique properties and potential applications in bioremediation and other fields. Besides, *Dechloromonas* is dominant in contaminated areas [46]. According to scientific reports, *Dechloromonas*, such as *D*. *agitate*, a particular species of bacteria, has the ability to release potassium from phyllosilicate minerals, which are commonly found in soil and rocks [47]. This finding could potentially have significant implications for soil fertility and plant production.

*Acidovorax* bacterial species belonging to the family Comamonadaceae, are gram-negative Betaproteobacteria. Among these species, *A*. *temperans* is a non-plant bacterium that has been isolated from human urine [48]. This bacterial strain is particularly interesting due to its potential implications for human health and disease. Besides, *Acidovorax* has been found in the microbiome associated with plant-parasitic nematodes, namely *Meloidogyne incognita* [49]. The same result was obtained in the present study. The mentioned bacterium has plant growth promotion potential, which impacts plant health and improves the production of tomatoes.

*Lactobacillus ruminis* is a type of bacteria that was initially discovered in human feces. Since then, it has been found in various other animals, including bovines, pigs, and horses, as reported by Yu et al. [50]. This particular strain of bacteria is known for its ability to produce lactic acid, which can help maintain a healthy gut environment. In a microbiome analysis of *Z*. *punctata*, *Lactobacillus* was found in the maize field of Limpopo Province, South Africa [12]. The same bacterial genus was found in the present study.

*Melissococcus plutonius* is a pathogenic bacterium that seriously threatens honeybees by causing brood disease. This bacterium has been identified as a major contributor to the decline in honeybee populations worldwide, making it a significant concern for beekeepers and the agricultural industry. The research conducted by Budge et al. [51] sheds light on the severity of this issue and highlights the need for effective measures to control the spread of this pathogen. It is imperative that beekeepers and researchers work together to develop strategies to mitigate the impact of *M*. *plutonius* on honeybee populations and ensure the sustainability of our agricultural systems. In a microbiome analysis of *Z*. *punctata*, *M*. *plutonius* was found in the maize field of Limpopo Province, South Africa [12]. The same bacterial species was found in the present study.

*Sphingomonads* are a diverse group of Gram-negative bacteria in diverse environments, such as aquatic and terrestrial habitats. They have been discovered in plant root systems, clinical specimens, and other sources, highlighting their versatility and adaptability. These bacteria play an important role in various ecological processes and have potential applications in bioremediation and biotechnology [52]. Research on the microbiome of *Caenorhabditis elegans* revealed that it contains *Sphingomonas* [53], known for its ability to break down complex organic compounds. This discovery sheds new light on the interactions between *C*. *elegans* and its microbiota. It may have implications for our understanding of the role of microbiomes in the health and survival of nematodes. The same bacterium was found in the present study.

It is important to mention that although the four most abundant bacterial species existed in all samples, their relative abundance varied. However, many less common bacteria were only found in some samples, indicating that *A*. *complexus* nematodes prefer certain bacterial species more strongly than others.

The study of the bacterial species associated with *A*. *complexus* nematodes provides valuable information about the intricate relationship between these tiny organisms and their environment. By analyzing the established type of nematode-bacteria association, researchers can understand the potential ecological consequences of this interaction. The findings of such studies can help us better understand the complex web of life and the interdependence of different species in the ecosystem. *Acrobeles complexus* can act as a vector for plant growth-promoting bacteria (PGRP) that enhance plant growth, such as *Arabidopsis* (*Sphingomonas* spp) and insect pathogens (*M*. *plutonius*). *Lactobacillus paraplantarum* bacteria have the ability to significantly influence the physiological processes of *A*. *complexus* or its microbiome by means of either antioxidant or antimicrobial activities [54].

Regarding the impact of *A*. *complexus* microbiome on the environment and agricultural activities, it should be noted that *A*. *complexus*, a free-living bacterivore, is known to feed on various types of bacteria, including *Dechloromonas*. This bacterium is essential for environmental management, as it plays a crucial role in reducing Nitrous Oxide (N_2_O) levels through denitrification in salty and stressed soil areas [55]. However, *A*. *complexus* does not prefer a specific type of bacteria, which means they can potentially reduce the number of *Dechloromonas*. This is particularly beneficial for agriculture, as N_2_O emission is a significant problem in the industry. Therefore, *A*. *complexus* can be an effective solution for reducing N_2_O levels and improving environmental management. Additionally, benzene is a toxic and carcinogenic compound that can contaminate ground and surface water, posing a serious threat to human health and the environment. Fortunately, *Dechloromonas* species, particularly *D*. *aromatica*, have been found to be effective in bioremediation of benzene-contaminated water. In situ bioremediation refers to the process of treating contaminated water or soil on site, using microorganisms like *D*. *aromatica* to break down harmful substances into harmless byproducts [56]. In addition, *D*. *aromatica* can also act as a bioindicator of soil contaminated with benzene, providing a useful tool for monitoring and assessing the extent of environmental contamination.

Besides, *A*. *complexus* is a species that feeds on *Lactobacillus*, a type of bacteria that plays a crucial role in regulating the soil’s organic matter and the biochemical cycle. *Lactobacillus* can detoxify hazardous chemicals, boost plant health, and have antibacterial properties, which is why it is widely used in biopreservation technology to ensure food safety [57, 58]. However, it has been observed that when the number of *Acrobeles* increases in the soil, there is a reduction in the number of *Lactobacillus* bacteria, which in turn creates an unsuitable environment for plant growth. Therefore, it is important to maintain a balanced population of these bacteria to ensure a healthy and fertile soil for plants to thrive.

Additionally, *A*. *complexus* feeds on *Clavibacter* (= *Corynebacterium*) and *Streptomyces*, two bacteria crucial to agriculture. *Clavibacter* is a harmful plant pathogenic bacteria that causes severe disease in tomatoes. For example, *C*. *michiganense* can cause up to 84% and 46% yield loss in commercial tomatoes in Canada and USA, respectively [59]. Therefore, reducing this bacterium in tomato fields through feeding by *A*. *complexus* positively impacts tomato production and increases the yield. In contrast, *Streptomyces* is a promising biocontrol agent of plant pathogens [60], and some species are plant pathogens, such as *S*. *scabies* [61].

In previous research, it was discovered that the population of *A*. *complexus* in tomato soil was found to be negatively correlated with the percentage of silt and clay in the soil while positively correlated with the amount of sand [6]. This result has been reaffirmed in the present study. This is because sand, which has more pores, provides a better habitat for the movement of *A*. *complexus*. This movement is crucial for the survival of the organism and its ability to thrive in the soil. Li et al. [62] indicated that pH had no effect on bacterivores nematode dynamics. The present study yielded the same outcome.

In conclusion, *A*. *complexus* is a soil-dwelling organism that coexists with various free-living and plant-parasitic nematodes in its habitat. The presence of these nematodes can lead to competition for food sources. However, *A*. *complexus* has a unique affinity towards certain bacteria, such as *Dechloromonas*, which can be considered a reliable indicator of soil health. This indicates that *A*. *complexus* has a specific preference for certain microorganisms that contribute to the soil’s overall health. The findings of our study revealed that the free-living *A*. *complexus* nematodes, which are commonly found in light soils of South African tomato fields, exhibit a remarkable preference for a diverse array of bacterial species, including plant pathogenic and beneficial bacteria. Therefore, it is crucial to comprehend the soil biodiversity and the biotic interactions that occur in these below-ground ecosystems to understand better how these organisms interact with one another and impact the health of the soil and the plants that grow in it. Our study highlights the significance of understanding the complex relationships between nematodes and bacteria in the soil and how these interactions can impact the health and productivity of crops. Additionally, a future study aims to delve deeper into the relationship between nematodes and plant pathogenic bacteria by comparing the microbiome of plants that are infected with those that are healthy. The study is expected to reveal the true nature of the interaction between the nematode and the plant pathogenic bacteria.

## Supporting information

S1 Fig Alpha diversity rarefaction plot based on ten iterations per sampling depth. (JPG)

S2 Fig Shannon entropy alpha diversity for bacterial communities associated with *A*. *complexus* nematodes collected from tomato fields. (JPG)

S3 Fig Relative frequency of the most abundant bacterial phyla associated with *A*. *complexus* nematodes collected from tomato fields. (JPG)

S4 Fig Relative frequency of the most abundant bacterial class associated with *A*. *complexus* nematodes collected from tomato fields. (JPG)

S5 Fig Relative frequency of the most abundant bacterial order associated with *A*. *complexus* nematodes collected from tomato fields. (JPG)

S6 Fig Relative frequency of the most abundant bacterial family associated with *A*. *complexus* nematodes collected from tomato fields. (JPG)

S7 Fig Relative frequency of the most abundant bacterial genus associated with *A*. *complexus* nematodes collected from tomato fields. (JPG)

S1 Table Measurements of *Acrobeles complexus* from Dalmada, Limpopo Province, South Africa. All measures in μm and the format: mean ± standard deviation (range).
(PDF)
