# Supplementary figures and images for: Bacterial communities associated with Acrobeles complexus nematodes recovered from tomato crops in South Africa
Source: PLoS One. 2024 Jun 6;19(6):e0304663. doi: 10.1371/journal.pone.0304663 (PMC11156337; doi:10.1371/journal.pone.0304663)

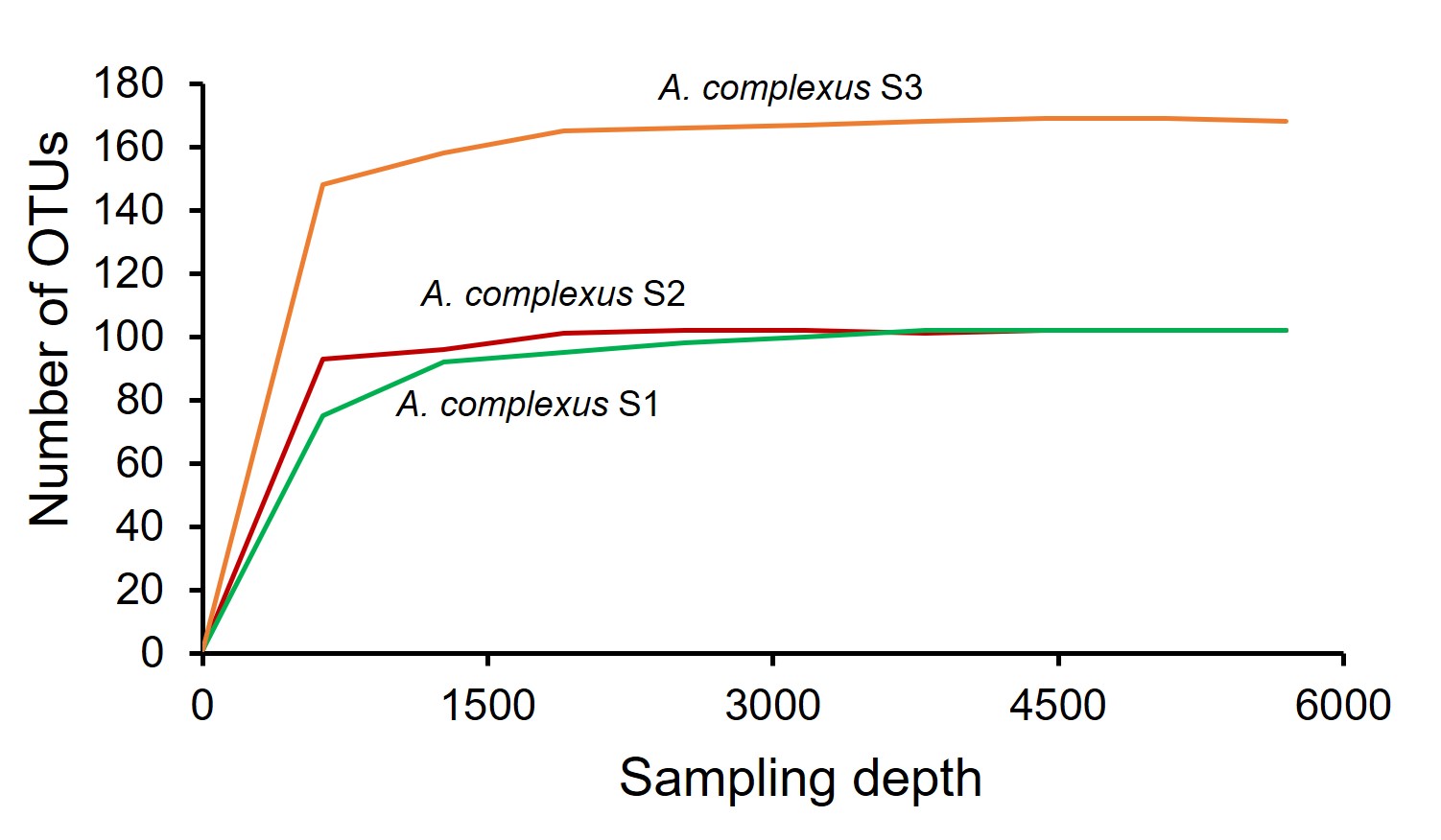

Supplement: S1 Fig — (JPG) [file pone.0304663.s001.jpg]

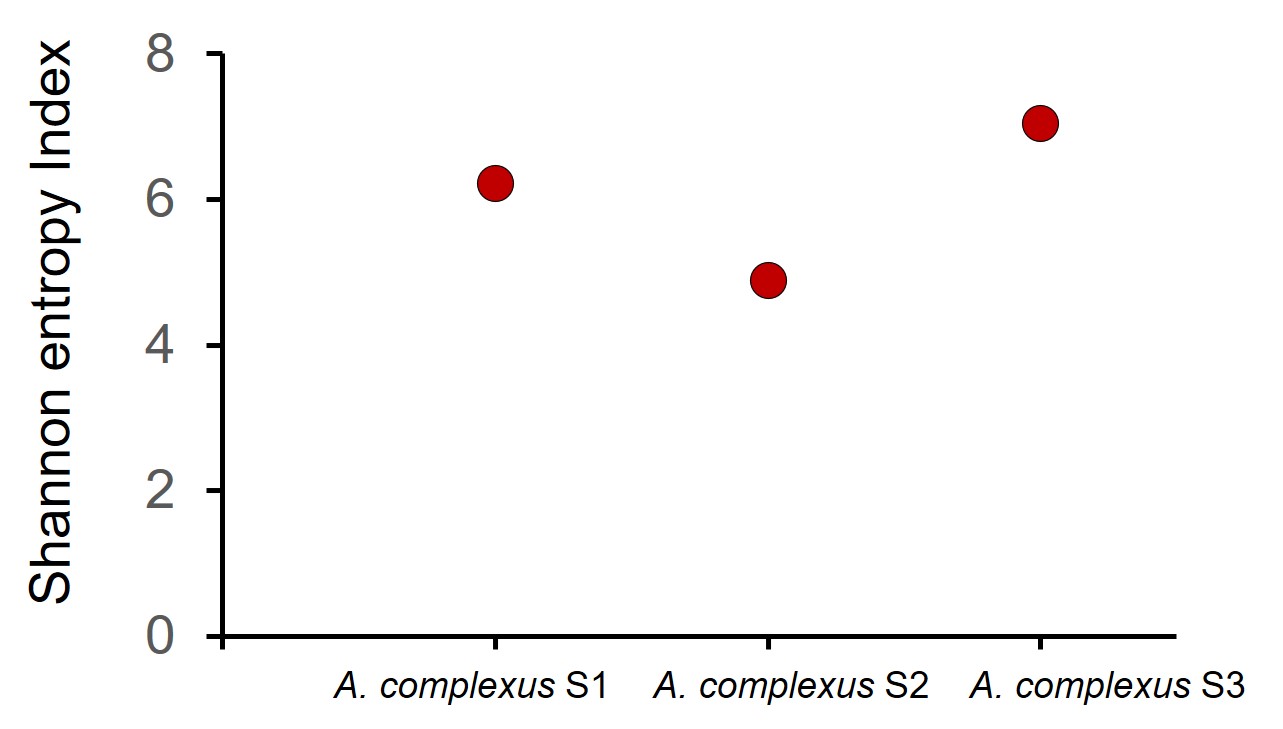

Supplement: S2 Fig — (JPG) [file pone.0304663.s002.jpg]

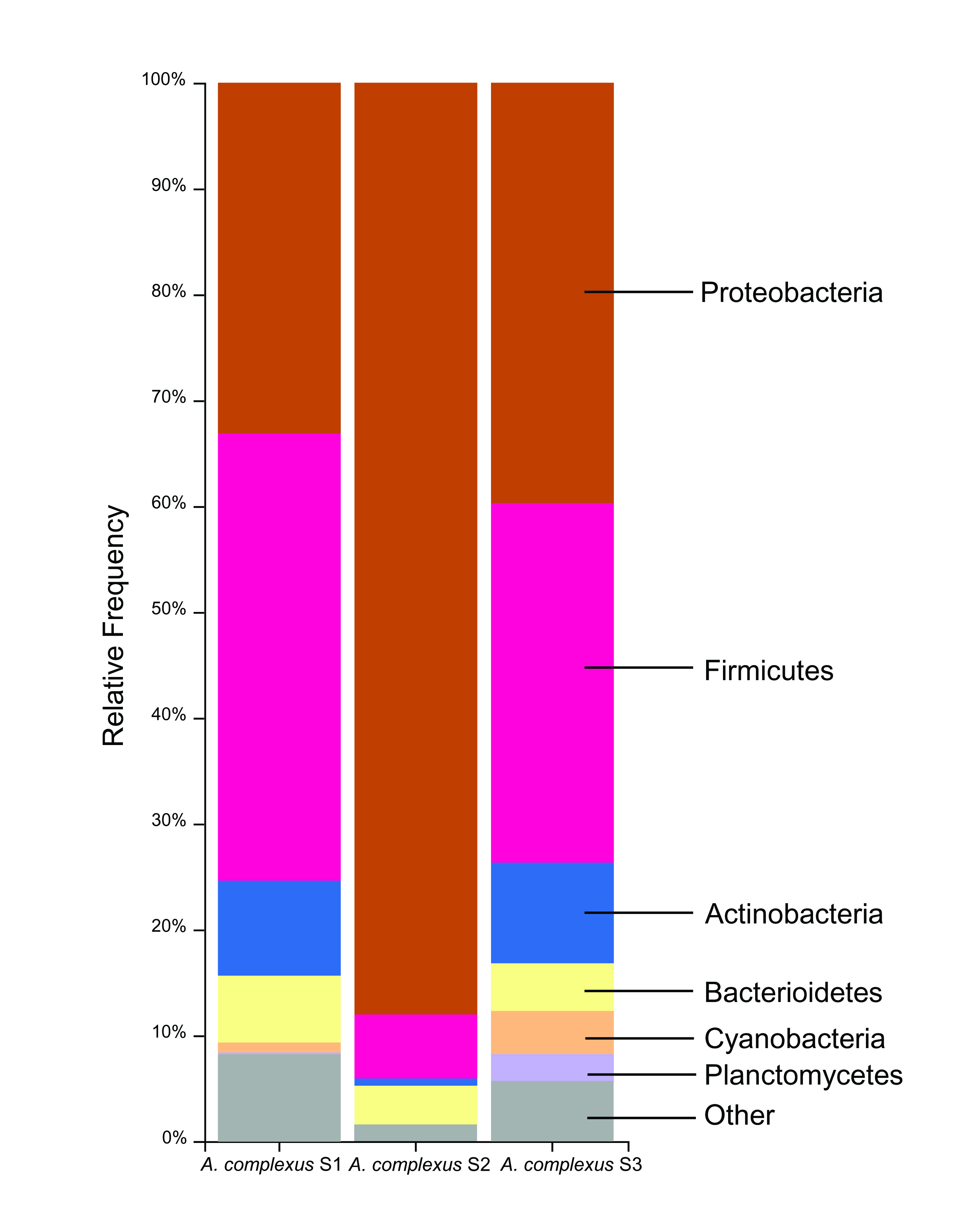

Supplement: S3 Fig — (JPG) [file pone.0304663.s003.jpg]

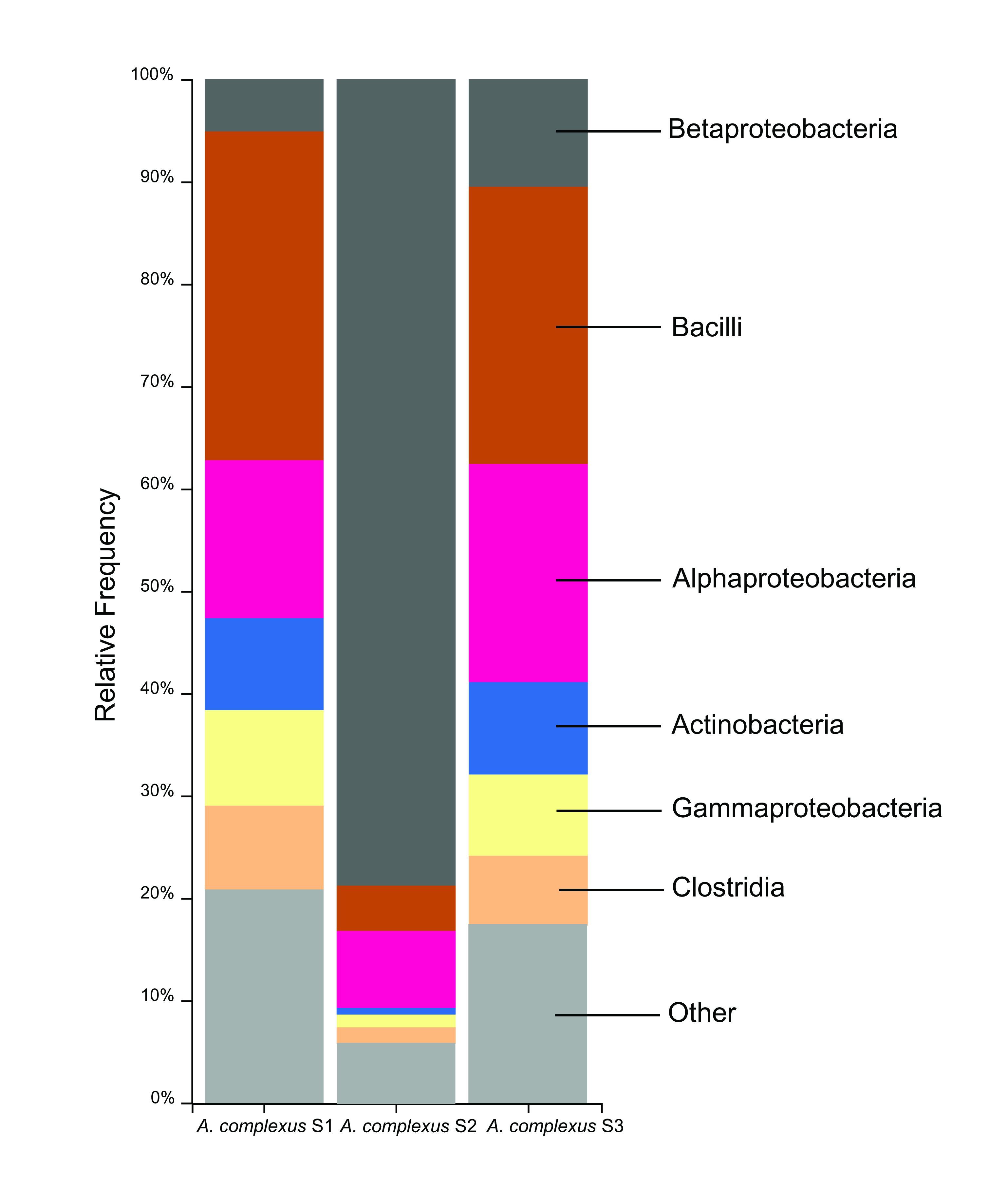

Supplement: S4 Fig — (JPG) [file pone.0304663.s004.jpg]

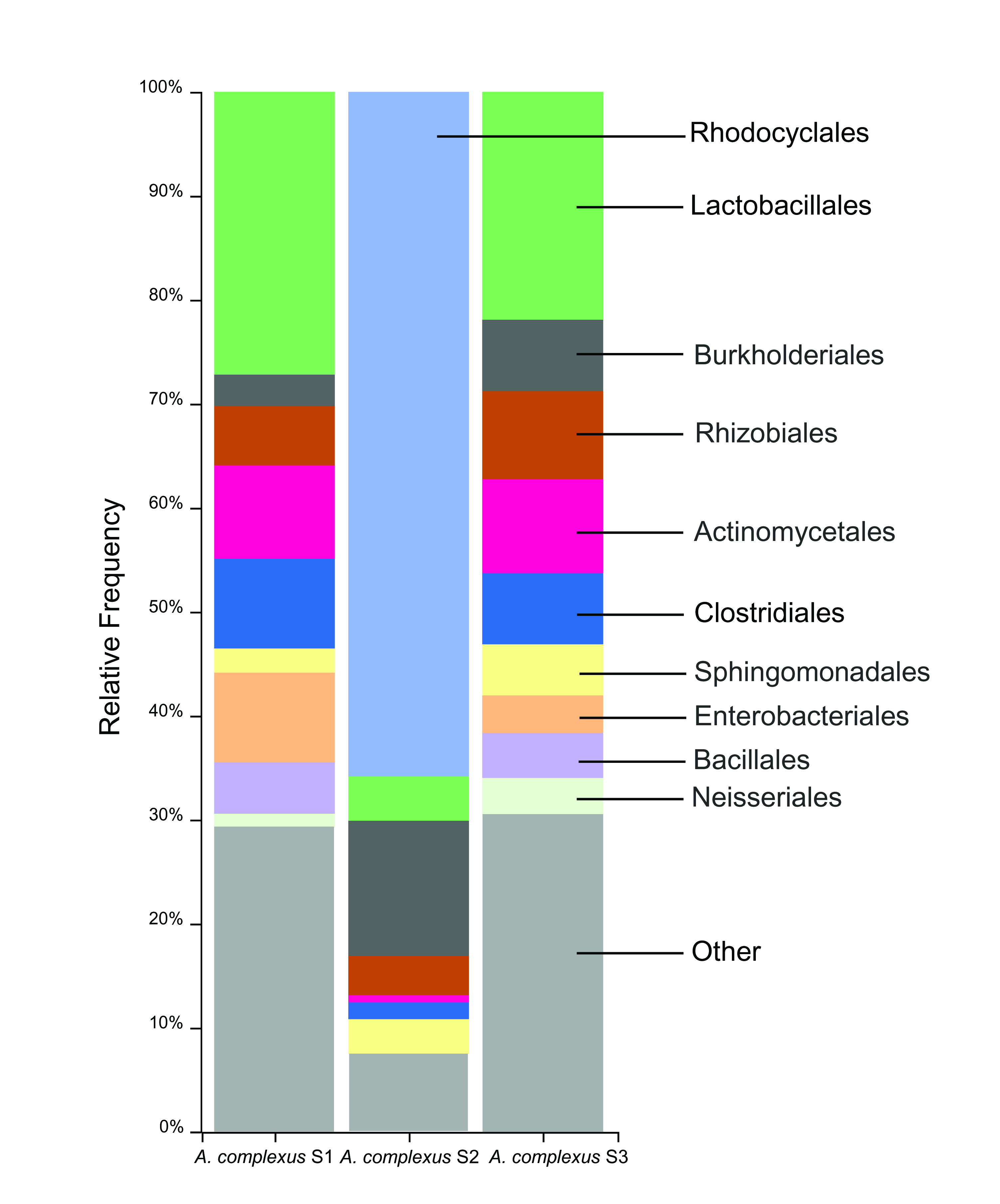

Supplement: S5 Fig — (JPG) [file pone.0304663.s005.jpg]

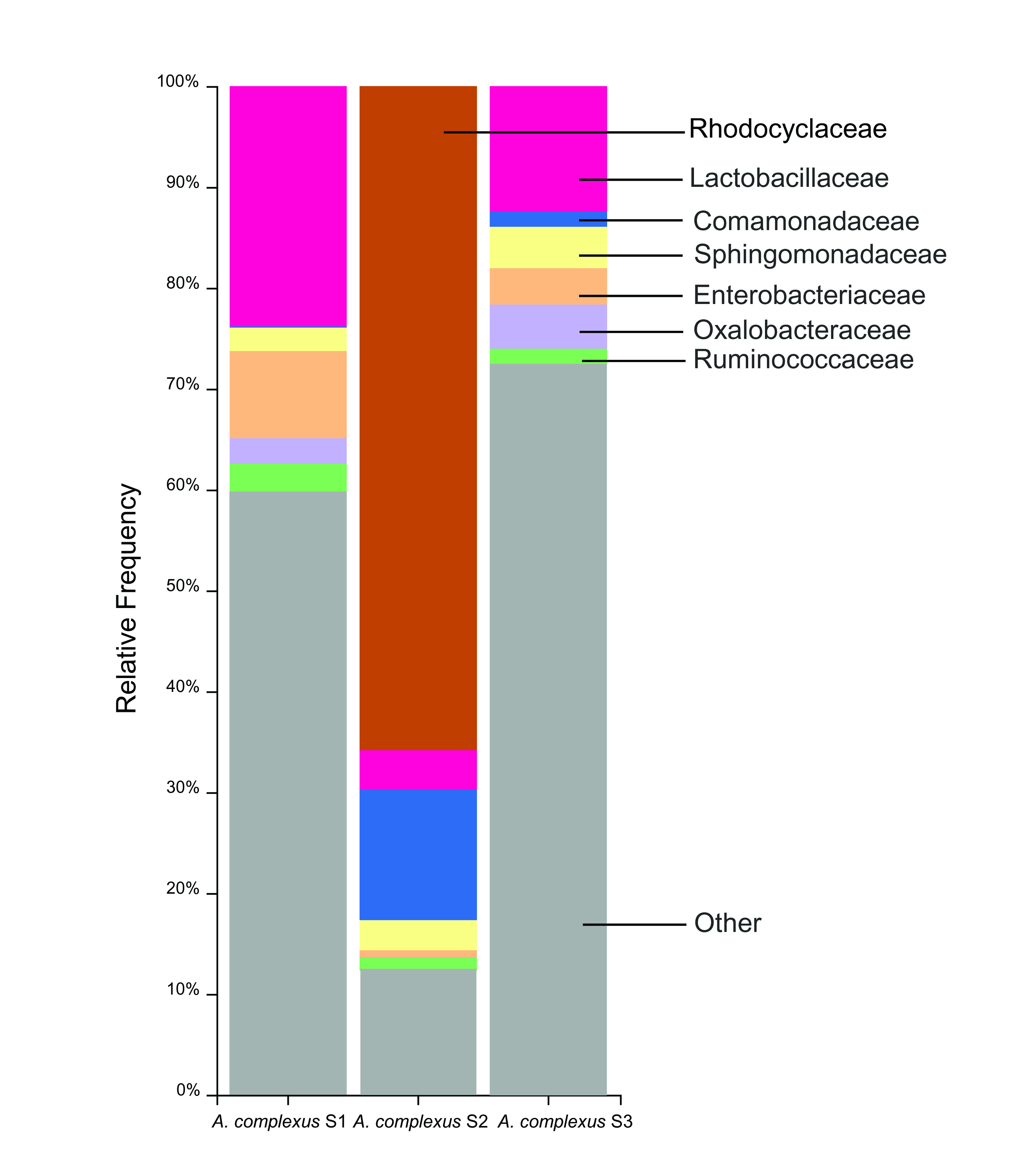

Supplement: S6 Fig — (JPG) [file pone.0304663.s006.jpg]

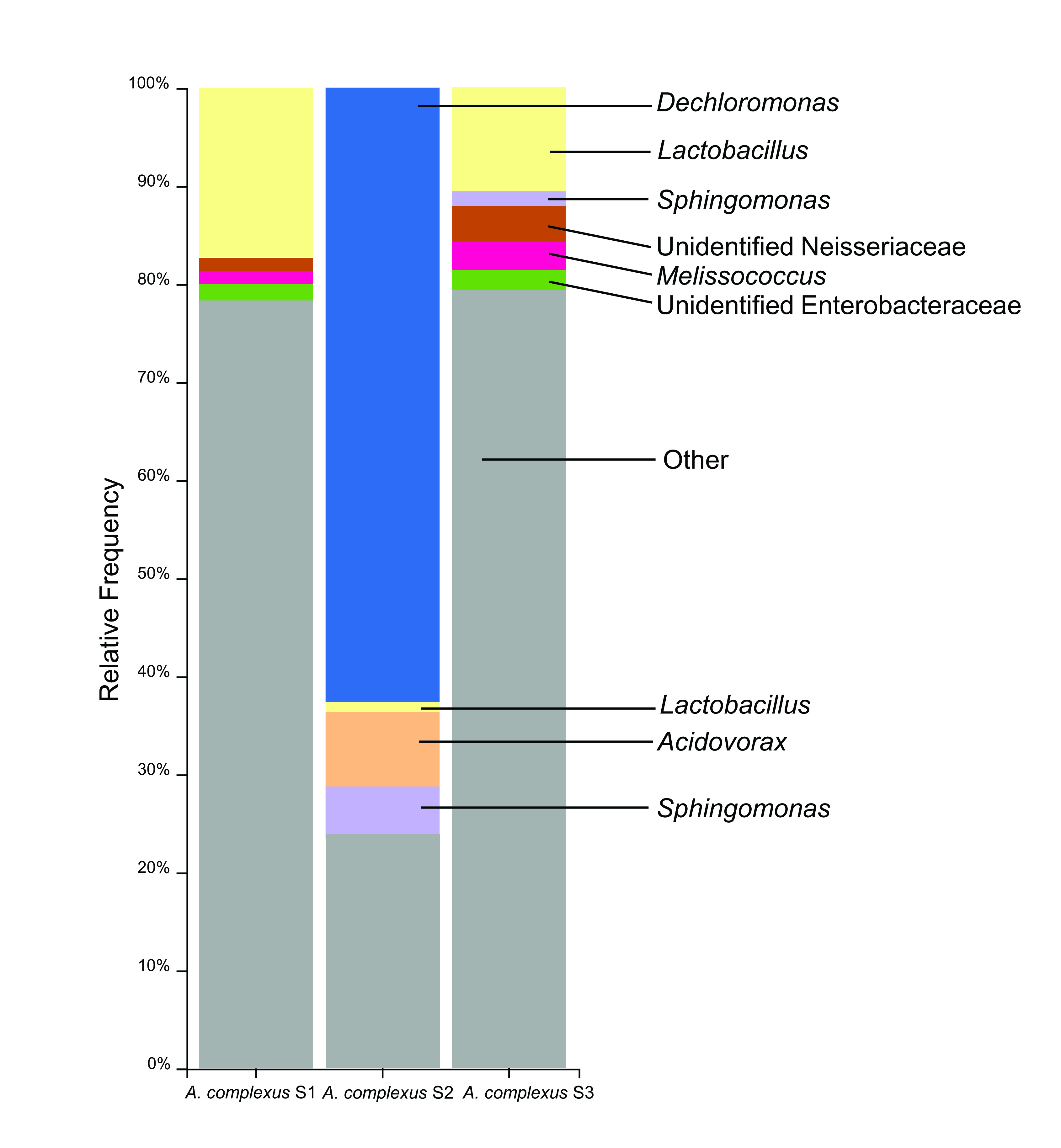

Supplement: S7 Fig — (JPG) [file pone.0304663.s007.jpg]
