# Supplementary material for: Bacterial communities associated with Acrobeles complexus nematodes recovered from tomato crops in South Africa
Source: PLoS One. 2024 Jun 6;19(6):e0304663. doi: 10.1371/journal.pone.0304663 (PMC11156337; doi:10.1371/journal.pone.0304663)
Supplement: S1 Table — All measures in μm and the format: mean ± standard deviation (range). (PDF) [file pone.0304663.s008.pdf]

Table S1. Measurements of *Acrobeles complexus* from Dalmada, Limpopo Province, South Africa. All measures in µm and the format: mean± standard deviation (range).

	3 ♀♀	♂
L	642 ± 21.5 (620-663)	585
a	20.9 ± 3.2 (17.4-23.8)	23.4
b	4.1 ± 0.2 (3.9-4.3)	3.8
c	9.3 ± 0.1 (9.3-9.5)	11.5
c'	3.8 ± 0.1 (3.7-3.9)	2.7
V	54.2 ± 0.7 (53-55)	-
Lip region width	13.7 ± 0.6 (13-14)	14
Labial probolae	12.7 ± 1.2 (12-14)	11
Stoma length	9.3 ± 0.6 (9-10)	8
Corpus length	86.3 ± 11.4 (77-99)	89
Isthmus length	36.3 ± 3.2 (34-40)	29
Terminal bulb length	26.0 ± 3.6 (23-30)	26
Nerve ring to anterior end	103.0 ± 4.0 (99-107)	92
Excretory pore to anterior end	107.3 ± 3.2 (105-111)	100
Neck length	157.3 ± 11.0 (150-170)	154
Body diameter at neck base	30.0 ± 6.1 (26-37)	25
Body diameter at mid body	31.3 ± 5.9 (27-38)	25
Body diameter at anus	18.0 ± 1.0 (17-19)	19
Vagina length	10.7 ± 1.2 (10-12)	-
Post vulval uterine sac length	72.0 ± 5.2 (69-78)	-
Rectum length	21.0 ± 6.2 (14-26)	29
Tail length	68.7 ± 2.1 (67-71)	51
Phasmid to anus	18.7 ± 1.2 (18-20)	-
Spicule length	-	43
Gubernaculum length	-	20
